# Harmful Algae Records in Venice Lagoon and in Po River Delta (Northern Adriatic Sea, Italy)

**DOI:** 10.1155/2014/806032

**Published:** 2014-02-10

**Authors:** Chiara Facca, Dagmar Bilaničovà, Giulio Pojana, Adriano Sfriso, Antonio Marcomini

**Affiliations:** ^1^Department of Environmental Sciences, Informatics and Statistics, Ca' Foscari University of Venice, Calle Larga Santa Marta 2137, 30123 Venice, Italy; ^2^Department of Philosophy and Cultural Heritage, Ca' Foscari University of Venice, Dorsoduro 3484/D, 30123 Venice, Italy

## Abstract

A detailed review of harmful algal blooms (HAB) in northern Adriatic Sea lagoons (Po River Delta and Venice lagoon) is presented to provide “updated reference conditions” for future research and monitoring activities. In the study areas, the high mollusc production requires the necessity to identify better methods able to prevent risks for human health and socioeconomical interests. So, an integrated approach for the identification and quantification of algal toxins is presented by combining microscopy techniques with Liquid Chromatography coupled with High Resolution Time of Flight Mass Spectrometry (HPLC-HR-TOF-MS). The method efficiency was first tested on some samples from the mentioned coastal areas, where *Dinophysis* spp. occurred during summer in the sites directly affected by seawaters. Although cell abundance was always <200 cells/L, the presence of Pectenotoxin-2 (PTX2), detected by HPLC-HR-TOF-MS, indicated the potential release of detectable amounts of toxins even at low cell abundance.

## 1. Introduction

Aquaculture and marine culture account for over a quarter of the world's seafood supply, but it is foreseen that this value will approach 50% by year 2030 [1]. As a consequence, aquaculture is now the fastest-growing food-producing industry in the world. While the aquaculture global supplies was 3.9% in the 1970s, it became 36% (51.7 million tonnes) in 2006 as weight of fish, crustaceans, and molluscs (FAO, Fisheries and Aquaculture Department, Report 2009). In the Italian marine waters the mollusc production from aquaculture activities sextupled from 1984 to 2005 and the prevailing cultured species, as production volumes, are currently mussels and clams (FAO, Fisheries and Aquaculture Department, Report 2009). In the northern Adriatic lagoons the clam catching and farming represent an important economic source: only in Venice lagoon approximately 800–1000 employees are regularly involved in clam farming on approximately 2000 ha which lead to a clam production of 25000 tonnes y^−1^ in 2007 with annual sale income of approximately € 52.000.000 [2].

According to this rapid growth, quality control for human health protection is strictly required, especially to prevent possible toxic effects arising from harmful algal blooms (HAB). The term “HAB” (HABs) is generally used to indicate algae that can cause a variety of deleterious effects on aquatic ecosystems, such as oxygen deficiency, clogging of fish gills, or poisoning of various organisms [3]. The oxygen deficiency occurs during the degradation of huge algal biomass and can cause mass mortality of benthic animals and fish. Approximately 70–80 microalgal species are already known to produce powerful toxins able to induce poisoning events. The economical impact of HAB events on the aquaculture industry has been never estimated in detail so far, but the economic loss of individual event in North America and Japan amounted to more than 10 million US$ (http://www.ioc-unesco.org/hab, accessed on August 20th, 2013). In addition to the potential damage to coastal ecosystems, including mortality of the fish stocks, toxins may be accumulated in shellfish and fish and then enter in the food webs, thus representing a serious health threat toward humans.

Although HAB events are natural phenomena, their impacts on the human health and on the coastal economy appeared to have increased in frequency, intensity, geographic distribution, and extension, since 1970 [4]. It is difficult to understand if HABs are actually rising or if it depends on a major scientific attention, paid to the problem over the last decades, concurrently to the significant increase in the use of coastal waters for aquaculture. However, the transport of dinoflagellate resting cysts in the ship ballast tanks and in the shellfish stock, as well as the alteration of environmental conditions, such as cultural eutrophication [5] and climate change, is suspected to be the main factor responsible for the spreading of HAB. Some recent experimental observations demonstrated that, due to recent climate alterations, the overall risk of harmful dinoflagellate and raphidophyte blooms in the Dutch coastal zone will increase rather than decrease in the future [6]. Recent poisoning events led to defining a hazard associated with HAB and prompted to understand more in detail occurrence and dynamics of HAB and to identify effective methods to prevent risks for human health and socioeconomical damage. Moreover, some algal toxins cannot be degraded even at temperatures >120°C [7], so thermal treatment (i.e., cooking) is not an effective measure to prevent poisoning. The only possibility to reduce the risk of consuming contaminated seafood is the application of intensive biological and chemical monitoring activities.

The taxonomic identification, usually performed by means of conventional optical microscopy and Scanning Electron Microscopy (SEM), can provide some indications on the presence of harmful algae but it is not sufficient to confirm or to foresee the occurrence of poisoning events. In fact, it has been observed that toxin production is influenced by many factors, that is, population density and environmental conditions [8] such as water column stratification or phosphorus limitation [9]. Hence, the microscopy observations, which are useful for preliminary identification, must be coupled with chemical analysis and/or functional or biochemical assay able to determine and quantify the toxin production.

The high economic relevance of aquaculture and the evidence of climate change suggest to closely monitor the phytoplankton community composition, especially in those areas where the production of shellfish is high. Beyond the monitoring of autochthonous species, the main risk for the future poisoning events is represented by the introduction and diffusion of alien species. Hence, an extensive bibliographic research was carried out with the double aim to describe the potentially harmful algal distribution in areas highly exploited for clam production and to resume the methods mainly used for the toxin identification. The former objective was to establish which algae may be responsible for poisoning events in the northern Adriatic lagoons and their past occurrence; the latter was to consider the pros and cons of the various techniques in order to choose the most suitable to quickly alert authorities in the case of potentially poisoning events. Eventually, an integrated approach for the identification of harmful algae, which applies a combination of optical and scanning microscopy with Liquid Chromatography coupled with High Resolution Time of Flight Mass Spectrometry (HPLC-HR-TOF-MS), is presented. The proposed approach was tested to investigate the occurrence and distribution of harmful algae in Venice lagoon and in Po River Delta, both located in the northern Adriatic Sea (Italy). To the best of our knowledge, the application of HR-TOF-MS has never been reported in the literature for the routine identification and quantification of algal toxins.

## 2. Materials and Methods

### 2.1. The Study Areas

**Figure 1:**
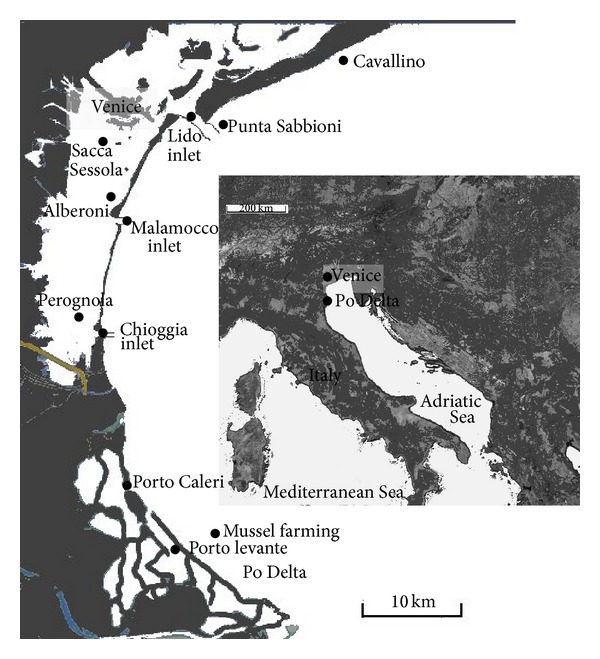
Map of the study area. The sampling sites are marked by black dots.

Venice lagoon (northern Adriatic Sea, Italy) with a total surface of ca. 549 km² is a very polymorphous shallow coastal environment which has not only a mean depth of 1 ± 0.3 m, but also large canals, connected with the sea through three inlets (Lido, Malamocco, Chioggia) 10–15 m deep (Figure 1). The tidal seawater flow through the three port inlets amounts approximately to a third of the total volume of the lagoon at each tidal cycle [10]. The mean astronomical tidal excursion in this lagoon is approximately ±31 cm but, under particular tidal events and/or meteorological conditions, values up to 170 cm above the mean sea level have been observed [11]. Freshwater inputs come from 12 main tributaries from a drainage basin of about 1850 km² [12], accounting for about 35 m³ s^−1^ y^−1^, with peak discharge under stormy conditions >344 m³ s^−1^ [13]. Po River is the major Italian river, which flows 640 km over the Padana Valley, a highly anthropized and industrialized area (approx 40 million equivalent inhabitants). Its delta is a complex system of flatlands and several lagoons where fish and clam farming is the main economic activity.

### 2.2. Materials

Okadaic acid (≥92%) and domoic acid (DA) (≥90%) were purchased from Sigma-Aldrich (Sigma-Aldrich Chemie GmbH, Stienheim, Germany) and solvents (acetonitrile, methanol, 2-propanol, and acetic acid) for HPLC ultragradient purity from Romil (Dublin, Ireland). Formaldehyde solution (~36% in water) and ammonium acetate buffer were from Fluka (Buchs, Switzerland). Water for chromatographic purposes was purified using a Milli-Q system (Millipore, Bedford, MS, USA). Individual stock solutions were prepared in 2-propanol and stored at 2°C at dark. The working standard solutions were monthly prepared by diluting the analytical standards in 2-propanol. Both working solutions and sample extracts were stored in brown glass vials (Agilent) at 2°C. Laboratory materials for analytical purposes were accurately cleaned with ammonium persulfate solution and then rinsed two times with 2-propanol before their use. GF/F glass fibre filters (Whatman, Landspert, NJ, USA) were precleaned by sonication with 2-propanol (2 h) and then gently dried overnight (12 h at 80°C). Because of their potential high hazard, all standards and application samples were handled with appropriate safety precautions.

### 2.3. Samplings

In order to record harmful algae occurrence, samplings were carried out from June 2009 to August 2009 along the shoreline, in the inlets and in proximity of clam farming in Venice lagoon and in two Po river delta lagoons (Figure 1). The sampling period was chosen in relation to the expected blooming months of *Dinophysis* [14], which may induce poisoning at 200 cells/L abundance [15]. For qualitative sampling of phytoplankton, a 20 *μ*m mesh phytoplankton net was used. The net was employed to verify the presence of rare harmful algae even below the detection limit of microscopic determinations as suggested by Penna et al. [16]. Aliquots of sample were preserved with formaldehyde solution (final concentration 2%), neutralised with hexamethylenetetramine for the taxonomic identification, and filtered on GF/C glass fiber filter (Whatman GF/C) in order to determine toxin occurrence by HPLC-HR-TOF-MS. For phytoplankton quantitative analyses surface water samples were instead collected and preserved with formaldehyde solution (final concentration 2%) neutralised with hexamethylenetetramine.

### 2.4. Phytoplankton Analyses

Qualitative samples were settled on the base chamber and observed on a light inverted microscope Axiovert 10 (Zeiss, Oberkochen, Germany) in order to identify species. The taxonomic identification was carried out using the texts of Avancini et al. [17], M. M. H. Peragallo and M. Peragallo [18], and Tomas [19, 20]. When potentially harmful algae were observed, quantitative analyses were further carried out in order to determine their cell abundance according to Utermöhl's method [21]. Pictures for archiving and comparison were taken by means of a Dino-Eye Piece Camera (AM323B, ANMO Electronics Corporation, CA, USA) installed on the light inverted microscope by means of a custom-made adapter. The taxonomic identification of the *Pseudonitzschia* sp. was performed also by Scanning Electron Microscopy (SEM) on a Philips Jeol 5600LV with EDX Oxford Instruments detector.

### 2.5. Extraction of Toxins from Algae Cells

Phytoplankton cell slurries were obtained by filtering approximately 50 L of sea water from each study area through a 20 *μ*m mesh phytoplankton net and were further filtered through 1.2 *μ*m glass fiber filter from Whatman. Filters were then gently dried and extracted with 10 mL of 80 : 20 vol% methanol : H_2_O mixture as reported by Blanco et al. [22] and sonicated in a Branson 5510 (Danbury, Connecticut, USA) sonication bath for 20 min. After sonication, extracts were 10 times concentrated by gentle evaporation under open air and centrifuged at 3500 rpm for 5 min. 500 *μ*L of the supernatant was then collected and transferred in 2 mL Teflon-lined screw capped brown glass vials stored at 4°C until their injection (10 *μ*L) in the HPLC-MS system.

### 2.6. HPLC-MS Analysis of Algal Toxins

The samples extracts were injected into an Agilent 1200 HPLC system (Palo Alto, CA, USA) using an Agilent G1329B autosampler. Detection and quantification of selected algal toxins were performed by using an Agilent G1969A High Resolution Time of Flight Mass Spectrometer coupled to the HPLC system via ElectroSpray Interface (ESI). Identification of analytes in real samples was carried out automatically by the Mass Hunter Data Analysis software based on retention time (±0.5 min of the corresponding standard) and on compound mass/charge ratio with <3 ppm mass accuracy. Such high resolution feature was possible by the use of a Dual-ESI interface which allows the continuous flow of a standard mixture for constant accurate mass calibration during the chromatographic run. The chromatographic separation of lipophilic toxins (OA, DTX1, PTX2) was performed using a Phenomenex (Torrance, CA, USA) Fusion stationary phase (100 × 2 mm, 2.5 *μ*m) protected by two guard columns containing the same stationary phase. The LC column temperature was set at 40°C by an Agilent G1316A thermostatted column compartment. The mobile phase (flow: 0.15 mL/min) was a mixture of methanol (A) and water (B), both containing ammonium acetate (10 mmol/L). A linear gradient elution, starting at 50% A and increased to 99% over 10 minutes, was employed. Nebulizing and drying gases of the TOF detector were nitrogen kept at 60 psig and 350°C, 10 L/min, respectively, while the main electric parameters were the following (negative ionization mode): fragmentor: 240V; skimmer: 100V; capillary voltage: 3750V; OCT1RT: 275V. Analysis of domoic acid in sample extracts was instead performed on a 150 × 2 mm, 5 *μ*m Phenomenex Gemini reversed-phase column. The mobile phase (flow: 0.1 mL/min) was a mixture of methanol (A) and water (B), both containing 10 mM ammonium acetate and 50 mM formic acid. A linear gradient elution, starting at 20% A and increased to 99% over 10 minutes, was employed and the column temperature was set to 30°C by an Agilent G1316A thermostated column compartment. Nebulizing and drying gases of the TOF detector were nitrogen kept at 60 psig and 350°C, 10 L/min, respectively, while the main electric parameters were (positive ionization mode) the following: fragmentor: 170V; skimmer: 60V, capillary voltage: 4000V, OCT1RT: 200V.

## 3. Results

### 3.1. Field Results on the Occurrence of HAB and Toxins in the Study Areas

**Figure 2:**
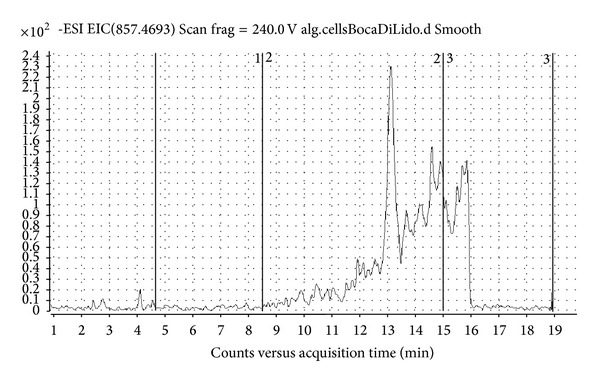
MS spectrum of PTX2 molecule and confirmation of exact formula generated from compound mass spectrum.

**Figure 3:**
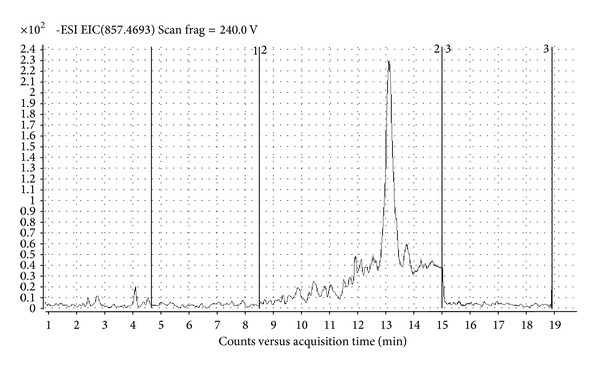
Selected ion monitoring HPLC-MS chromatogram of PTX2.

*Dinophysis caudata *Saville-Kent, *D. mitra* (F. Schütt) T. H. Abé, and *D. sacculus* Stein were present in most of the areas directly influenced by seawaters (Table 2), but not in the inner parts. Even though *Dinophysis *spp. occurrence was revealed, the chemical analysis highlighted that they were unable to produce detectable amounts of okadaic acid and DTX1. However, in cell extract collected at Lido inlet molecular ion [M-H]^−^ with m/z 857.4671 has been detected corresponding to molecular ion of PTX2 molecule. MS-TOF software confirms presence of PTX2 by generating exact formula from compound mass spectrum (Figure 2). Quantification of the found toxin by selected ion monitoring HPLC-MS chromatogram of PTX2 (Figure 3) was not possible due to the lack of pure toxin. Although *D. caudata* Saville-Kent has been found also at Chioggia and Malamocco inlets and Po Delta sites, presence of PTX2 was not detected, probably due to lower abundance of algal cells. In addition to *Dinophysis* spp., also other nontoxic Dinophyceae, such as *Ceratium* spp., were poorly abundant or absent in the inner part of the lagoons and near the clam farming areas. In those areas, in fact, the community was mainly constituted by benthic or epiphytic diatoms (*Amphora* spp., *Cocconeis* spp., *Gyrosigma* spp., and *Navicula* spp.), which were resuspended from the shallow bottoms, by colonial diatom species such as *Skeletonema *sp. or *Chaetoceros* spp. and by small flagellates.

The relative abundance of *Prorocentrum cordatum* with respect to the entire phytoplanktonic community was <3% and the absolute abundance did not trigger poisoning events.

**Figure 4:**
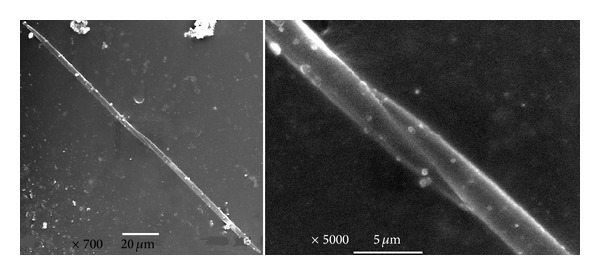
SEM images for *Pseudonitzschia pseudodelicatissima*. The pointed and tapering ends are highlighted.

Among potentially harmful algae, some species of the genus *Pseudonitzschia *were found in all the sites. At least 11 species of the genus have already been proved to be toxic but a correct taxonomic identification of the species would require more sophisticated technique such as Scanning Electron Microscopy (SEM) [20]. To avoid a wrong identification, samples were prepared for the SEM analysis (Figure 4). Some structural details of *Pseudonitzschia delicatissima *(Cleve) Heiden, which is known to produce domoic acid, cannot be clearly recognised with the conventional optical microscopy and may be confused with *Pseudonitzschia pseudodelicatissima *(Hasle) Hasle, for which toxin production has seldom been reported [23]. The latter microalga is characterized by more pointed and tapering ends [20]. In Figure 4 it is possible to verify that the species was *P. pseudodelicatissima *(Hasle) Hasle, so it did not represent a risk. Moreover, the cell relative abundance was generally <3.5%; hence, the potentially toxin production would be negligible, even in presence of a toxic strain of *P. delicatissima*. Eventually, domoic acid was not detected in the phytoplankton extracts, screened by HPLC-MS HR-TOF, so confirming that *Pseudonitzschia* sp. found in the lagoon of Venice and in Delta was not toxic.

## 4. Discussion

### 4.1. Past Records of Harmful Algae in the Study Areas

In the North-western Adriatic Sea the first poisoning event due to algal toxins was recorded in 1989 along the Emilia-Romagna coast [15]. Since then, particular attention has been paid to the phytoplankton community composition and the local public health authorities started to plan monitoring programs in order to prevent the sale of contaminated seafood. Current Italian legislation indicates the use of Yasumoto's test to verify the bivalve poisoning level.

In Venice lagoon, harmful algae have been rarely reported and they were observed only at the inlets and seldom in the inner lagoon waters. In Table 1 the full list of records is reported. For each taxon, the year during which it was observed, the associated toxins and the consequent syndrome are reported. The potential to produce toxin of *Prorocentrum cordatum *(ex *minimum*) (Ostenfeld) Dodge is still matter of investigation, so the reference to a review is supplied [24]. The distribution of *Dinophysis*, which represents the most hazardous genus in the Venetian area, was yet described in 1947-49. The species *D. sacculus* Stein and *D. tripos* Gourret were found occasionally during the flowing tide in the Chioggia inlet, whereas *D. caudata *Saville-Kent, which is euryhaline, was more frequent reaching up to 100 cells/L, in August-October, and it was observed also in the inner areas [25]. Afterwards, in the 1970s, only *D. sacculus* Stein was found occasionally in the marine stations [26]. In the 1980s *Dinophysis* spp. abundance was always negligible and <1%. Moreover, they were recorded in the seawards stations and again never in the inner areas [27]. In the 1990s, *D. caudata* Saville-Kent appeared to be frequent but occasional [27], although the increase of records may depend more on the sampling frequency than on an actual enhancement of the species occurrence as poisoning events have never been observed in those areas. In general, the highest abundance of *Dinophysis* spp. was recorded in late summer-autumn. The other genus, which is often present in Venice lagoon waters, is *Pseudonitzschia* but in this case not all the species are toxic and SEM determination becomes necessary for their correct identification. Most of the authors have recorded *Pseudonitzschia* in the lagoon waters since the 1970s (Table 1), but it is considered an occasional and seldom abundant species [26], mainly occurring in the flow waters and near the inlets [27]. *Amphora coffeaeformis *(C. Agardh) Kützing, with toxicity still questioned as only a strain from Canada was found to produce domoic acid, while other strains were nontoxic [28], appeared to be constant in the lagoon waters also in the inner areas [27]. Abundance around 10% was recorded for *Prorocentrum cordatum *in aquaculture areas and near fishing ponds [27].

In Po lagoons the occurrence of potentially harmful algae has always been sporadic and their abundance of no concern for aquaculture activities [27]. In some cases, in the lagoons connected with Po River and Adriatic Sea, *P. cordatum *reached up to 2 × 10^6^ cells/L [27], but the farming activity was never stopped. Evidence for toxin production was seldom demonstrated but it has often been associated with human poisoning via shellfish ingestion, so it must be considered potentially toxic, pending for further investigations [24]. Moreover, due to its high capability in adaptation to different environmental conditions and its fast growth, it could represent a serious threat for human health.

However, some critical situations, due to massive *Alexandrium tamarense* (Lebour) E. Balech (Saxitoxin producer) blooms (2–4 × 10^6^ cells/L), were recorded in the fishing ponds, where aquaculture is intensive and the water renewal is man-regulated [47]. These events were observed in mid-summer, in closed basins with high waste inputs, but the blooms did not occur in the adjacent and connected ponds where the water renewal was higher.

The massive input of freshwater from Po River may probably explain the poor abundance of dinoflagellates, which are generally limited by low salinity values. Until now, North of Po River Delta, poisoning biotoxin episodes have not been reported even though some harmful algae (such as *Dinophysis* spp.) have been commonly recorded at very low (<100 cells/L) concentration level in the coastal seawaters [14].

### 4.2. Resume of Methods for Toxin Identification

An accurate quantitative determination of algal toxins can be performed by means of chemical or biochemical techniques. The first step in the purification and analysis is usually cell disruption (lysis), which is one of the most critical steps affecting the yield of searched toxins. The method used may vary depending on the type of cell: for soft animal tissues, homogenizer probes, vortex mixers, or blenders are used [7, 48–50]; for algal cells, sonication as homogenization method is recommended [51]. Sonication disrupts nonspecifically cell-surface barriers and transports molecules across both cell membranes and cell walls [52]. However, when algal cells are disrupted in a nondenaturing media, homogenization efficiency is incomplete [51, 53]. Thus, sonication of isolated algal cells should be carried out in presence of organic solvent.

Chemical determination of algal toxins is usually performed by HPLC-MS after solvent extraction [7, 54, 55]. Toxins have different molecular characteristics (i.e., polarity and ionization preferences), and thus different chromatographic separation and MS detection conditions have to be developed and applied. Toxins can be lipophilic such as the okadaic acid (OA) group, the azaspiracid (AZA) group, the yessotoxin (YTX) group, and the pectenotoxin (PTX) group, which are usually extracted from biological samples with 100% or 80% methanol [7, 49, 56], or can be polar such as domoic acid (DA), usually extracted with water and methanol in various proportions [50, 57, 58]. Saxitoxins (STXs) and their derivates require extraction by acidified water or alcohol media [59–61]. The HPLC-MS technique permits to identify and quantify with good accuracy toxins of known structures, and allows the simultaneous determination of more toxins in the same sample. Single quadrupole or triple quadrupole instruments are usually employed [50, 55]. Recently, more sophisticated instruments such as quadrupole ToF (Q-ToF) have been proposed for the structural identification [49, 62]. The typical ionization mode for OA, DTX1, and DTX2 molecules is negative [7, 49, 54], while domoic acid, STX, and AZA group are preferably ionized under positive mode [49, 54, 55, 57, 60]; PTX group is ionized both under positive and negative mode [57, 63–65]. Other methods based on HPLC coupled with fluorescence or UV have been also reported [58, 66]. Advanced mass spectrometer detectors such as High Resolution Time Of Flight (HR-TOF) and Orbitrap are highly compound specific being capable of accurate mass full-scan measurements, thus confirming the structure of the searched compounds and overcoming their intrinsic lower sensitivity. Moreover, in comparison with triple quadrupole instruments, the accurate mass measurement allows the correct identification of high molecular weight toxins, such as palytoxin, which can be subjected to structure modifications nondetectable by low resolution instruments [67].

The biological determinations are usually performed according to Yasumoto's test: acetone extracts of mussel digestive glands are intraperitoneally injected to 3 mice. The limit of detection of such mouse assay is approximately 0.8 g equiv. OA g^−1^ digestive gland in shellfish [68]. However, such method is rather expensive, time-consuming, and sometimes unqualified to determine in detail which toxins or which modifications produce the syndrome and is in contrast with recent European Commission indications. The European Parliament is, in fact, revising the Directive 86/609/EEC on the protection of animals used for experimental and other scientific purposes, in order to promote improvements in the welfare of laboratory animals and to further foster the development of alternative methods (http://ec.europa.eu/environment/chemicals/lab_animals/nextsteps_en.htm, accessed on August 20th, 2013).

The use of biochemical or functional assays as alternative methods to the animal testing has been recently proposed. Biosensors are projected in order to generate an electrical signal proportional to the binding between the toxin and an acceptor (antibody or receptor). However, such method has been validated only for saxitoxin and analogs. On the other hand, a functional assay uses the mechanism of action of the overall toxin group, so it can determine the presence of the group and the total toxicity compared to the reference activity of the most representative compound of each group [54, 69, 70]. This method is rather cheap and allows to analyze a large number of samples at once, providing a direct interpretation of the potential toxicity. However, it should be coupled with chemical methods, which identify the toxin pool chemical profile [69, 70].

### 4.3. Current Records of Harmful Algae in the Study Areas

In Venice lagoon, the presence of dinoflagellates has always been negligible (Table 1; [38]) probably due to the complex hydrodynamism, since approx 1/3 of the entire water volume is exchanged at each tidal cycle [10], and to a high water turbidity [71]. In fact, the performed sampling sessions demonstrated that *Dinophysis caudata *Saville-Kent, *Dinophysis mitra* (F. Schütt) T. H. Abé, and *Dinophysis sacculus* Stein were present during summer period only in the areas directly influenced by seawaters, such as Venice inlets and Po Delta. Quantitative observations demonstrated that their abundance was always significantly below the conventional threshold (~200 cells/L) triggering poisoning events. The same algae were not detected in the inner Venice lagoon, as previously reported by the literature. It was already observed that *Dinophysis* growth is favored by water column stability [8, 14], which is an unlikely condition in shallow lagoons undergoing significant tidal cycle. The presence of an algal toxin (Pectenotoxin-2, PTX2) was anyway detected and structurally identified for the first time by HPLC-HR-TOF-MS in samples from Venice lagoon inlets, so indicating the potential release of toxins in detectable amounts from *Dinophysis *spp. though at low cell concentration levels. Luckily, the toxin concentrations were very low and, at that time, they did not represent a risk. However, monitoring activities are constantly necessary to protect human health.

Even though it was not observed in 2009, *P. cordatum* may form dense blooms in coastal and estuarine systems (see references in [72]) and its toxicity has been widely observed, but it is not known which toxins are actually produced. Bivalve mortality was observed in laboratory together with a massive migration of hemocytes into the stomach and intestine to protect the tissues from exposure to the toxic algae [73].

## 5. Conclusions

The HAB events occurred in Venice and Po Delta lagoons have been reviewed in order to collect “updated reference conditions” for future research and monitoring activities. In addition, an integrated approach for the structural identification and quantification of harmful algae, applying a combination of techniques such as optical and scanning microscopy (OM, SEM), and Liquid Chromatography coupled with High Resolution Time of Flight Mass Spectrometry (HPLC-HR-TOF-MS), is presented. The proposed approach has been successfully applied to the investigation of harmful algae occurrence and distribution in the above-mentioned coastal areas.

Further sampling sessions are planned to gain a deeper insight into the possible occurrence of the examined and other toxic algae in the study areas, as well as to evaluate possible future scenarios under remarkable climate change.

## Figures and Tables

**Table 1:** Harmful algae records in the lagoon of Venice.

	Year	Reference	Syndrome	Toxins
*Alexandrium* spp.	1993	[29]	Paralytic shellfish poisoning (PSP)	Gonyautoxins (GTX1, GTX2, GTX3 and GTX4); Saxitoxin (STX).
	2000–2002	[30]		
	2003	Facca unpublished		
	2000-2001	[31]		

*Dinophysis* spp.	1947–1949	[25]	Diarrhetic shellfish poisoning (DSP)	Okadaic acid and related congeners (DTX1, DTX2); pectenotoxins (PTX2)
		[32]		
	1971	[26]		
	1988-1989	[33]		
	1991/1992	[34]		
	1993-1994	[35]		
	1980/1998	[31]		

*Prorocentrum lima *	1983 2003	[36] Facca, unpublished	Diarrhetic shellfish poisoning (DSP)	DSP-type toxins; okadaic acid and related congeners (DTX1, DTX2); Pectenotoxins (PTX2, PTX2sa)

*Prorocentrum cordatum* ex *Prorocentrum minimum *	1988-1989	[33]		
	1990-1991	[37]		
	1990-1991	[34]		
	1993-1994	[35]		
	1993; 1998	[29]	[24]	
	2000–2003	Facca, unpublished		
	1998–2007	[38]		
	1980/1992-1993/ 1998–2002	[31]		

*Pseudonitzschia * spp.		[32]	Amnesic shellfish poisoning (ASP)	Domoic acid
	1970	[39]		
	1971	[40]		
	1986	[41]		
	1971-1972	[26]		
	1975–1980	[42, 43]		
	1979-1980	[44, 45]		
	1988-1989	[33]		
	1990-1991	[37]		
	1990-1991	[46]		
	1993-1994	[35]		
	1997–2002	[42, 43]		
	2000–2003/2005	Facca, unpublished		
	1998–2007	[38]		
	1979/1992-1993	[31]		

**Table 2:** Harmful algae occurrence in the study area. Cell abundance is expressed as cell/L.

Species	*Dinophysis caudata *	*Dinophysis mitra *	*Dinophysis sacculus *	*Noctiluca scintillans *
Authors	Saville-Kent	(Schutt) Abé and Balech	Stein	(Macartney) Kofoid and Swezy
Lido inlet (Venice)	37	<37		75
Chioggia inlet (Venice)	<30			<30
Malamocco inlet (Venice)	<30			
Perognola (Venice)				
Sacca Sessola (Venice)				
Alberoni (Venice)				
Punta Sabbioni (Sea-Venice)			<37	
Cavallino (Sea-Venice)			100	
Porto Caleri (Po Delta)				
Porto Levante (Po Delta)	<20			
Porto Levante (Po Delta)				
Mussel farming (Sea-Po Delta)	20		20	20
